# Transposon insertion causes cadherin mis-splicing and confers resistance to Bt cotton in pink bollworm from China

**DOI:** 10.1038/s41598-019-43889-x

**Published:** 2019-05-16

**Authors:** Ling Wang, Jintao Wang, Yuemin Ma, Peng Wan, Kaiyu Liu, Shengbo Cong, Yutao Xiao, Dong Xu, Kongming Wu, Jeffrey A. Fabrick, Xianchun Li, Bruce E. Tabashnik

**Affiliations:** 1 Key Laboratory of Integrated Pest Management On Crops in Central China, Ministry of Agriculture, Hubei Key Laboratory of Crop Disease, Insect Pests and Weeds Control, Institute of Plant Protection and Soil Fertility, Hubei Academy of Agricultural Sciences, Wuhan, 430064 China; 2 State Key Laboratory for Biology of Plant Diseases and Insect Pests, Institute of Plant Protection, Chinese Academy of Agricultural Sciences, Beijing, 100193 China; 3 Hubei Insect Resources Utilization and Sustainable Pest Management Key Laboratory, Huazhong Agricultural University, Wuhan, 430070 China; 4 School of Life Science, Central China Normal University, Wuhan, 430079 China; 5 Agricultural Genomics Institute at Shenzhen, Chinese Academy of Agricultural Sciences, Shenzhen, 518120 China; 6 USDA, ARS, U.S. Arid Land Agricultural Research Center, Maricopa, Arizona 85138 USA; 7 Department of Entomology, University of Arizona, Tucson, Arizona 85721 USA

**Keywords:** Pathogens, Molecular biology

## Abstract

Transgenic crops producing insecticidal proteins from *Bacillus thuringiensis* (Bt) are cultivated extensively, but rapid evolution of resistance by pests reduces their efficacy. We report a 3,370-bp insertion in a cadherin gene associated with resistance to Bt toxin Cry1Ac in the pink bollworm (*Pectinophora gossypiella*), a devastating global cotton pest. We found the allele (*r15*) harboring this insertion in a field population from China. The insertion is a miniature inverted repeat transposable element (MITE) that contains two additional transposons and produces two mis-spliced transcript variants (*r15A* and *r15B*). A strain homozygous for *r15* had 290-fold resistance to Cry1Ac, little or no cross-resistance to Cry2Ab, and completed its life cycle on Bt cotton producing Cry1Ac. Inheritance of resistance was recessive and tightly linked with *r15*. For transformed insect cells, susceptibility to Cry1Ac was greater for cells producing the wild-type cadherin than for cells producing the *r15* mutant proteins. Recombinant cadherin protein occurred on the cell surface in cells transformed with the wild-type or *r15A* sequences, but not in cells transformed with the *r15B* sequence. The similar resistance of pink bollworm to Cry1Ac in laboratory- and field-selected insects from China, India and the U.S. provides a basis for developing international resistance management practices.

## Introduction

Crops genetically engineered to produce insecticidal proteins from *Bacillus thuringiensis* (Bt) have been widely adopted for pest control, with a cumulative total of over 930 million hectares of Bt crops planted globally from 1996 to 2017^1^. These Bt crops kill some major pests, but cause little or no harm to humans and most other organisms^2–6^. The benefits of Bt crops include pest suppression, decreased insecticide use, and enhanced biological control^2–4^. However, increasingly rapid evolution of resistance by insect pests has reduced these benefits^7–10^.

The most common mechanism of resistance to Bt toxins is reduced binding of toxins to midgut receptor proteins including cadherin, alkaline phosphatase, aminopeptidase N, and ATP-binding cassette transporters^11–17^. The insertion of transposons, which can confer resistance to chemical insecticides, can also cause resistance to Bt toxins by disrupting genes encoding Bt receptor proteins^18–26^.

This study focuses on a novel cadherin allele that harbors a transposon and is linked with resistance to Bt toxin Cry1Ac in pink bollworm (*Pectinophora gossypiella*), a devastating global pest of cotton. Pink bollworm has been exposed extensively to Bt cotton producing Cry1Ac in the world’s top three cotton-producing countries: China, India, and the U.S.^27–29^. In China and the U.S., Cry1Ac has remained effective against pink bollworm for the past two decades^7,27,29^. In India, however, this pest evolved practical resistance to Bt cotton producing Cry1Ac alone, then to Bt cotton producing both Cry1Ac and Cry2Ab^7,28,30–32^.

Previous work shows that resistance to Cry1Ac in pink bollworm is associated with mutations in the *PgCad1* gene, which encodes a cadherin protein that binds Cry1Ac in susceptible larvae^30,33–35^. Fourteen *PgCad1* resistance alleles have been identified: *r1-r4* in lab-selected strains from the U.S.^33,36^, *r5-r12* in field-selected populations from India^30^, and *r13-r14* in a field-selected strain from the Yangtze River Valley of China^35^.

In this study, we detected a novel *PgCad1* allele (*r15*) while screening pink bollworm collected in 2013 from field populations in the Yangtze River Valley of China. Here we characterize this allele in terms of its DNA sequence, including insertion of three nested transposons. We also analyzed the resistance in a strain homozygous for this allele, including inheritance, cross-resistance to Cry2Ab, as well as survival and other life history traits on Bt cotton. In addition, we used transfected insect cells to determine the effects of the mutant allele on cadherin protein movement within cells and the susceptibility of cells to Cry1Ac.

## Results

### Identification of the *r15* cadherin allele

We started pink bollworm strain JL46 by pairing a field-collected male (#46) from Jianli in the Yangtze River Valley with a resistant female (cadherin genotype *r1r1*) from the lab-selected AZP-R strain from Arizona, U.S.A. Survival of their F_1_ offspring was 47% at the diagnostic concentration of Cry1Ac (10 μg Cry1Ac protoxin per ml diet), suggesting that the field-collected male carried one recessive resistance allele at the cadherin locus. We used a series of crosses, DNA screening, and selection with Cry1Ac to generate strain JL46 (Fig. S1). We discovered that strain JL46 had a novel cadherin allele, which we name *r15*, following the nomenclature convention for *PgCad1* resistance alleles^30,33,35^.

**Figure 1:**
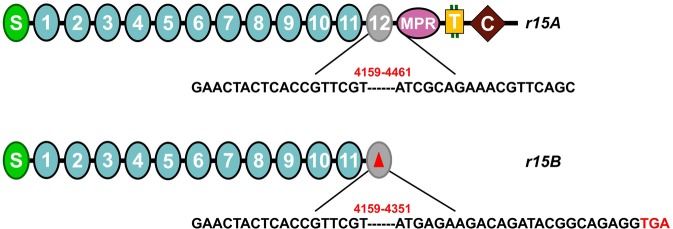
Predicted cadherin protein in pink bollworm strain JL46. The amino-terminal membrane signal sequence (S), cadherin repeats (1–12), membrane proximal region (MPR), transmembrane region (T), and cytoplasmic domain (C) are shown. Red numbers indicate deletions in the cDNA from *r15A* (303 bp) and *r15B* (193 bp). The red triangle indicates the truncation of the protein predicted from *r15B* because of the premature stop codon (red letters TGA).

**Figure 2:**
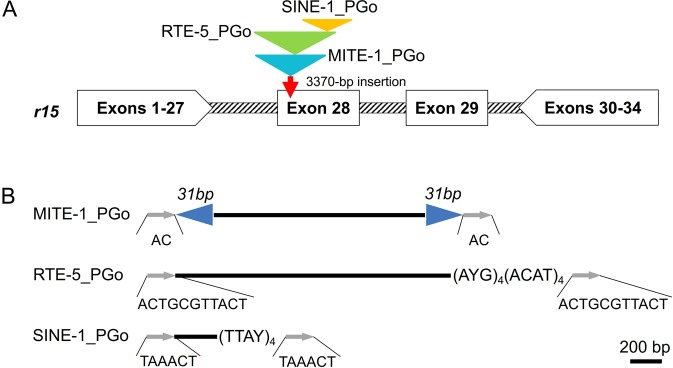
Map of the *PgCad1 r15* mutation. (**A**) *r15* allele of *PgCad1*. The 3,370-bp insertion in exon 28 contains remnants of three nested transposons, each shown as a colored triangle. (**B**) Details of the three nested transposons. The primary structure of each transposon is drawn to scale. Gray arrows show target site duplications (TSDs) (sequences below the arrows). Blue arrowheads represent the 31-bp terminal inverted repeats (TIRs). (AYG)_4_(ACAT)_4_ in RTE-5_PGo and (TTAY)_4_ in SINE-1_PGo are microsatellite repeats.

Sequencing cDNA of *PgCad1* from JL46 revealed that *r15* has two different transcripts. Transcript *r15A* (GenBank Acc# KY814704) has a 303-bp deletion (4,159–4,461) encoding a PgCad1 protein that lacks 101 amino acids, 88 from cadherin repeat twelve (CR12) and 13 from the membrane proximal region (MPR) (Figs 1 and S2). Transcript *r15B* (GenBank Acc# KY814705) has a 193-bp deletion (4,159–4,351) that creates a frameshift and introduces a premature stop codon at amino acid 1,394 (Figs 1 and S2). Sequencing of corresponding gDNA showed *r15* has a 3,370 bp insertion (1,166–4,535) in exon 28 (GenBank Acc# KY814708), which is not in the wild-type (*s*) allele (GenBank Acc# MH266779) (Fig. 2). Alignment of cDNA sequences indicates that the 3,370-bp insertion causes skipping during pre-mRNA splicing that yields two aberrant transcripts: *r15A* missing exons 28 and 29, and *r15B* missing only exon 28 (Fig. S3).

### Allele-specific PCR

Based on the 3,370-bp insertion in *PgCad1* gDNA in the *r15* allele relative to the *s* allele from the susceptible strain APHIS-S, we designed one pair of primers that amplified gDNA from *r15* but not from *s*, and another pair that amplified gDNA from *s*, but not from *r15* (Table S1). By testing individuals with both pairs of primers, we could distinguish among the three genotypes *r15r15, ss*, and *r15s* (Fig. S4). All of the larvae tested had the expected bands indicating *r15r15* for JL46, *ss* for APHIS-S, and *r15s* for their F_1_ progeny (total n = 90 larvae, 30 of each type).

### Characterization of three nested transposons in *r15*

By direct sequencing of the 3,370-bp insertion in *r15*, we discovered three new transposable elements (TEs) in exon 28 of *PgCad1* (Figs 2 and S5). Based on their similarity to known TEs, we name these *MITE1_PGo* (a miniature inverted repeat transposable element), *RTE-5_PGo* (a retrotransposable element type 5), and *SINE-1_PGo* (a short interspersed nuclear element). These three TEs are nested: *RTE-5_PGo* within *MITE1_PGo* and *SINE-1_PGo* within *RTE-5_PGo* (Fig. 2).

*MITE1_PGo* (1,342 bp), which apparently was inserted first into exon 28, is AT rich (71%). It has 31-bp terminal inverted repeats (TIRs) (left TIR = ATATGGGCTATTATTTTATTTTGGGTCCCAT) flanked by 2-bp (AC) target site duplications (TSDs), and lacks coding potential (Figs 2 and S5).

*RTE-5_PGo* is 1,751 bp (1,941–3,680, 3,964–3,974) and flanked by 11-bp TSDs (ACTGCGTTACT) (Figs 2 and S5). A CENSOR search^37^ against the Repbase (http://www.girinst.org/repbase/index.html) shows that the first 503 bp of this TE corresponds to an apurinic-apyrimidic endonuclease (AP ENDO) domain and shares 76% identity with *RTE-4_PPo* (431–935) from *Papilio polytes*^38^ (Table S2). The final 982 bp of this insert corresponds to an apparent reverse transcriptase (RT) domain, sharing 77% identity with *RTE-5_DPl* (1,719–2,719) from *Danaus plexippus*^39^ (Table S2). Further alignments show that sequences corresponding to bases 243–612 and 634–1,611 (Fig. S6) share 71% and 75% identity with *RTE-5_DPl* (361–731 and 1,719–2,714), respectively. This indicates an internal deletion of about 1,000 bp occurs within this TE. It no longer has an intact open reading frame (ORF) because indels and other mutations yield frameshifts and pre-mature stop codons. Nonetheless, it does encode an interrupted and internally deleted endonuclease-reverse transcriptase with 50% amino acid identity within the putative AP ENDO domain and 74% identity within the RT domain from RTE-5_DPl (Fig. S7), as well as 80% identity (data not shown) in the RT domain with the *Bombyx mori* non-LTR retrotransposon BmRTE-d08 (GenBank accession nos. FJ265549.1). Its 3′ untranslated region (UTR) is short and ends with four AYG trinucleotide repeats followed by four ACAT tetramer repeats (Figs 2 and S5), the typical features found in the members of the RTE clade^40^.

*SINE-1_PGo* is 283 bp (3,681–3,963) (Figs 2 and S5). This TE is flanked by 6-bp (TAAACT) TSDs, lacks coding potential, and has four TTAY repeats at its 3′ end (Figs 2 and S5).

### Inheritance of Cry1Ac resistance

Based on the concentration of Cry1Ac killing 50% of larvae (LC_50_), JL46 had 290-fold resistance relative to APHIS-S (Table 1). The responses to Cry1Ac for F_1_ larvae from the two reciprocal crosses between JL46 and APHIS-S were similar (Table 1), which indicates inheritance of resistance to Cry1Ac was autosomal (no sex linkage or maternal effects). Survival at the diagnostic concentration was 83% for JL46 and 0% for both APHIS-S and F_1_ progeny from the two reciprocal crosses (n = 72 larvae for each strain and reciprocal cross). At this concentration of Cry1Ac, the value for the dominance parameter *h* was 0 for both reciprocal crosses, indicating inheritance of resistance was completely recessive.

**Table 1:** Responses to Cry1Ac of pink bollworm larvae from a resistant strain (JL46), a susceptible strain (APHIS-S), and their hybrid F_1_ progeny.

Strain	Slope (SE)^a^	LC_50_ (95% FL)^b^	RR^c^
APHIS-S	3.78 (0.336)	0.097 (0.048–0.132)	
JL46	2.41 (0.506)	28.0 (22.4–37.3)	290
JL46♀ × APHIS-S♂	3.34 (0.305)	0.448 (0.397–0.497)	4.6
JL46♂ × APHIS-S♀	2.23 (0.223)	0.548 (0.463–0.648)	5.6

^a^Slope of the concentration-mortality line with its standard error in parentheses.

^b^Concentration killing 50% with 95% fiducial limits in parentheses, in μg Cry1Ac per ml diet.

^c^Resistance ratio, the LC_50_ for JL46, JL46♀ × APHIS-S♂ or JL46♂ × APHIS-S♀ divided by the LC_50_ for APHIS-S.

### Little or no cross-resistance to Cry2Ab

The LC_50_ of Cry2Ab was 30% higher for JL46 than APHIS-S, but this difference between strains is not statistically significant based on the conservative criterion of non-overlap of the 95% fiducial limits of the LC_50_ values (Table S3). Hence, JL46 had little or no cross-resistance to Cry2Ab.

### Genetic linkage between resistance to Cry1Ac and *PgCad1*

We used genetic linkage analysis to test the hypothesis that Cry1Ac resistance in JL46 is linked with the *r15* allele (Table S4). We generated five backcross families, each from a single-pair cross between a JL46 female and an F_1_ male (JL46 × APHIS-S). For the backcross progeny on untreated diet, the mean percentage of larvae that were *r15r15* (49%) did not differ significantly from the 50% expected under random segregation (one-sample *t*-test, df = 4, P = 0.56, Table S4). For the backcross progeny on diet treated with the diagnostic concentration of Cry1Ac, all 105 survivors were *r15r15*. The proportion of *r15r15* survivors was significantly higher on treated diet than that on control diet (Fisher’s exact test, P < 10^−18^). These results indicate Cry1Ac resistance is tightly linked with *r15* in JL46.

### Survival and other life history traits on Bt and non-Bt cotton

Larval survival on Bt cotton bolls was significantly higher for JL46 (13.0%) than APHIS-S (0.0%) (t-test, t = 28.7, df = 4, P < 0.0001, Table S5). However, on non-Bt cotton bolls, larval survival did not differ significantly between JL46 (27.1%) and APHIS-S (31.1%) (t-test, t = −2.7, df = 4, P = 0.052, Table S5). Relative survival, calculated as larval survival on Bt cotton divided by larval survival on non-Bt cotton, was significantly higher for JL46 (48.0%) than for APHIS-S (0.0%) (t-test, t = 14.0, df = 4, P < 0.0001).

On non-Bt cotton bolls, pupal weight did not differ significantly between JL46 and APHIS-S, but the time to develop from neonate to pupa was significantly longer for JL46 (16.7 days) than that for APHIS-S (15.0 days) (Table 2). For JL46, the time to pupation was also significantly longer on Bt cotton (21.8 days) than on non-Bt cotton (16.7 days), and pupal weight on Bt cotton (11.9 mg) was significantly less than on non-Bt cotton (14.3 mg) (Table 2). For JL46, both survival from neonate to adult and the proportion of adults that were female were significantly lower on Bt cotton than that on non-Bt cotton (Fisher’s exact test, P < 0.0001 for each trait). By contrast, no difference occurred between Bt cotton and non-Bt cotton for eggs laid per female (t-test, t = 1.6, df = 5, P = 0.17) and hatching rate of eggs (t-test, t = 0.21, df = 12, P = 0.84) (Table 3). Based on all of the above measures, the net reproductive rate^41^ for JL46 was four times higher on non-Bt cotton than that on Bt cotton, indicating incomplete resistance of JL46 to Bt cotton.

**Table 2:** Time to pupation and pupal weight for pink bollworm on Bt and non-Bt cotton bolls.

Strain	Cotton type	Number of pupae	Time to pupation (days)	Pupal wt. (mg)
APHIS-S	Non-Bt	70	15.0 ± 0.2a	13.7 ± 0.4a
JL46	Non-Bt	60	16.7 ± 0.2b	14.3 ± 0.5a
JL46	Bt	23	21.8 ± 0.5c	11.9 ± 0.6b

Values are means ± SE. Different lower case letters within columns indicate significant differences between treatments based on ANOVA followed by Tukey’s HSD.

**Table 3:** Life history traits of resistant pink bollworm strain JL46 on Bt and non-Bt cotton bolls.

Trait	Bt	Non-Bt	Bt/non-Bt
Neonate to adult survival	0.11	0.23	0.48
Proportion of females	0.36	0.44	0.82
Eggs per female	133 ± 30	209 ± 34	0.64
Hatch rate	0.83 ± 0.04	0.82 ± 0.02	1.01
Net reproductive rate^a^	4.4	17.3	0.25

^a^Net reproductive rate = neonate to adult survival × proportion of females × eggs per female × hatch rate^41^.

### PgCad1 cellular trafficking and susceptibility to Cry1Ac in transformed cells

**Figure 3:**
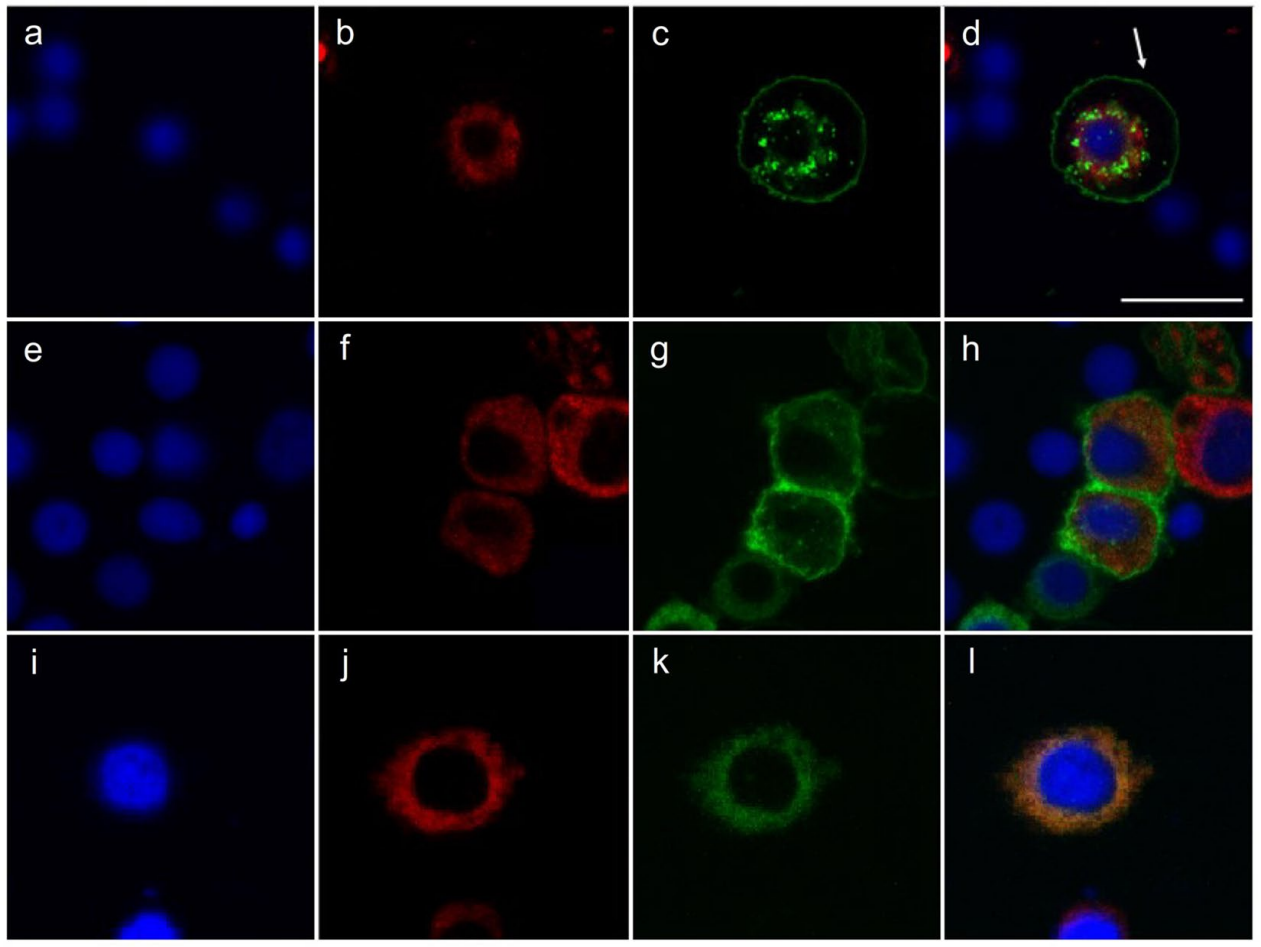
Cellular localization of PgCad1 proteins within Hi5 cells. Hi5 cells transfected with pIE2-sPgCad1-GFP (**a**–**d**), pIE2-r15APgCad1-GFP (**e**–**h**), or pIE2-r15BPgCad1-GFP (**i**–**l**). Nuclei stained with Hoechst 3342 are shown in blue, dsRED-labeled endoplasmic reticulum shown in red, and GFP-labeled PgCad1 fusion proteins are shown in green. Superimposed images from (**a**–**c**) are shown in (**d**), from (**e**–**g**) in (**h**), and from (**i**–**k**) in (**l**). The arrow in (**d**) indicates cell membrane. Bar = 20 μm.

We used Hi5 cells transfected with expression vectors to produce recombinant PgCad1 proteins fused with a green fluorescent protein (sPgCad1-GFP, r15APgCad1-GFP, or r15BPgCad1-GFP) (Fig. 3). Transfection efficiencies (mean % ± SD) did not differ significantly between sPgCad1-GFP (69 ± 15%), r15APgCad1-GFP (72 ± 7%), and r15BPgCad1-GFP (61 ± 8%) (one-way ANOVA, P = 0.47).

Hi5 cells producing PgCad1 proteins were co-transfected the pIE2-DsRed2-ER vector that specifically labels the endoplasmic reticulum (ER) with a red fluorescent protein (Fig. 3). Whereas sPgCad1-GFP and r15APgCad1-GFP localized primarily with the cell membrane (Fig. 3a–h), r15BPgCad1-GFP localized with the ER (Fig. 3i–l). Immunoblots with transfected cell extracts confirmed that the recombinant fusion proteins produced by the transformed cells had the expected molecular weights (sPgCad1-GFP = 253 kDa; r15APgCad1-GFP = 240 kDa; and r15BPgCad1-GFP = 209 kDa) (Fig. S8).

**Figure 4:**
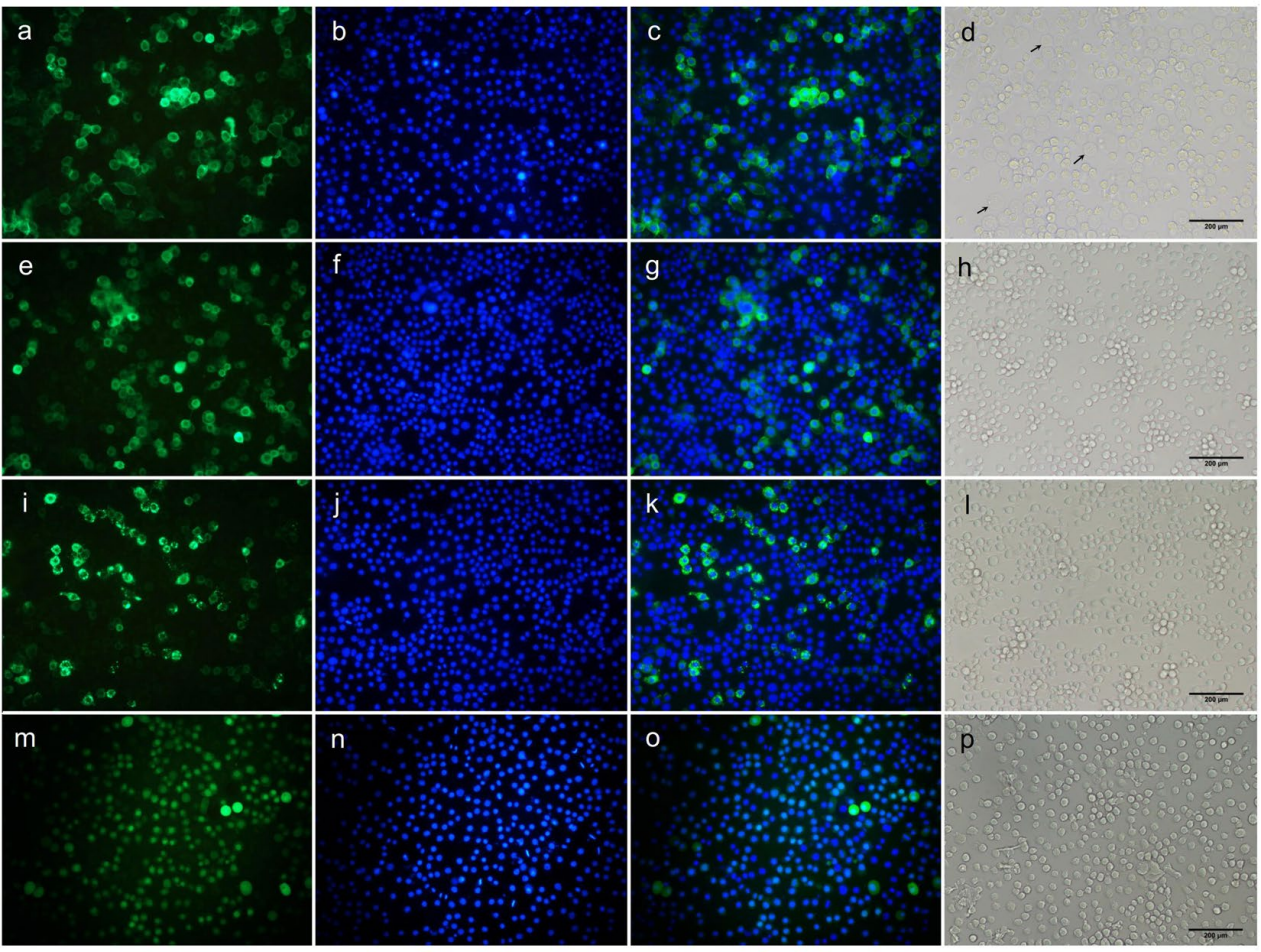
Susceptibility to Cry1Ac of Hi5 cells producing PgCad1 proteins. Hi5 cells transfected with pIE2-sPgCad1-GFP (**a**–**d**), pIE2-r15APgCad1-GFP (**e**–**h**), pIE2-r15BPgCad1-GFP (**i**–**l**) or the empty vector pIE2-GFP (**m**–**p**) were treated with Cry1Ac (10 μg Cry1Ac per ml for cells producing sPgCad1-GFP and 40 μg Cry1Ac per ml for r15A- and r15BPgCad1-GFP and GFP cells) and observed for swelling using fluorescence microscopy. Nuclei stained with Hoechst 3342 are shown in blue and PgCad1-GFP fusion proteins are shown in green. Superimposed images from (**a**,**b**) are shown in (**c**), from (**e**,**f**) in (**g**), from (**i**,**j**) in (**k**) and from (**m**,**n**) in (**o**). Arrows in (**d**) indicate representative swollen cells. Bars shown in (**d**,**h**,**l**,**p**) = 200 μm.

Treatment of the transformed Hi5 cells with Cry1Ac caused swelling and cell lysis in cells producing sPgCad1-GFP (Fig. 4a–d) but not in cells producing r15APgCad1-GFP (Fig. 4e–h), r15BPgCad1-GFP (Fig. 4i–l), or GFP (Fig. 4m–p). The concentration of Cry1Ac causing swelling in 50% of the sPgCad1-GFP cells (EC_50_) was 7.3 μg/ml (95% FL = 6.2 to 8.4). In contrast, no swelling was observed for cells producing r15APgCad1-GFP or r15BPgCad1-GFP at the highest concentration tested (40 μg Cry1Ac/ml).

## Discussion

Understanding the genetic basis of pest resistance is increasingly important for sustaining the efficacy of Bt crops^29,42^. Here, we discovered and characterized a novel mutant cadherin allele (*r15*) of the pink bollworm gene *PgCad1* from a field population in China. The *r15* allele contains a 3,370-bp insertion in exon 28 that results in disrupted pre-mRNA splicing and produces two aberrant transcript variants (*r15A* and *r15B*). Both variants result from exon skipping, with the *r15A* transcript lacking exons 28 and 29, and the *r15B* transcript missing only exon 28.

Previous results provide two other examples of transposable element insertion into *PgCad1* associated with mis-splicing and resistance to Cry1Ac^26,30^. In the *r3* allele found in some laboratory-selected strains from Arizona, insertion of a 4,739-bp intact, active non-LTR retrotransposon causes loss of exon 21^26^. The *r5* allele detected in a field-selected larva from India harbors a 3,120-bp insertion that shares sequence similarity with several transposable elements^30^.

Mis-splicing of cadherin pre-mRNA that does not involve TE insertion is also associated with resistance to Cry1Ac in pink bollworm from the U.S., India, and China^30,33,35^. For example, the 126-bp *r2* deletion from Arizona leads to mis-splicing and complete loss of exon 16^33^. In India, the *PgCad1 r10* allele results in three alternatively spliced transcripts^30^. Such alternative exon usage is also present in pink bollworm from China, as shown here with the *r15A* and *r15B* transcript variants. The *PgCad1 r13* mutation from China also involves mis-splicing, in which the 314-bp deletion results in complete loss of exon 31^35^.

Although the *r15* mutation is novel, the resistance in JL46 is similar to that of other Cry1Ac-resistant strains from the U.S. and China that have cadherin resistance alleles (e.g., *r1–r4* and *r13*)^33,35,36,41,43,44^. Like JL46, these strains have recessive inheritance of high levels of resistance to Cry1Ac and little or no cross-resistance to Cry2Ab. Although their larvae can survive on Bt cotton producing Cry1Ac, the resistance is incomplete, as indicated by reduced performance on Bt cotton relative to non-Bt cotton. Here we found the net reproductive rate on Bt cotton relative to non-Bt cotton for JL46 (0.25) was similar to that of the AQ47 strain from China (0.16) that harbors the *r13 PgCad1* allele^35^ and with U.S. resistant strains carrying *r1-r3* alleles (0.35)^43^.

Similar to previous results with the *r13* allele of *PgCad1* and the *mHaCad* allele of *Helicoverpa armigera*^35,45^, our data with the r15B protein suggest that failure of cadherin to move to the cell membrane causes resistance. By contrast, we found that the r15A protein did occur on the cell membrane, which implies a different mechanism of resistance, such as reduced toxin binding.

Pink bollworm resistance to Bt cotton remains a threat, especially where non-Bt cotton refuges are scarce. In India, farmers have generally not planted such refuges, pink bollworm resistance to Bt cotton producing Cry1Ac and Cry2Ab is a serious problem, and no Bt traits are currently available to control resistant insects^28,30–32^. In the Yangtze River Valley of China, the frequency of pink bollworm resistance to Cry1Ac initially went up, then declined from 2011–2015, corresponding with increased abundance of non-Bt cotton generated by the rise in planting of F_2_ hybrid cotton^29^. Nonetheless, the continued presence of cadherin resistance alleles in field populations demonstrated here and previously^35^ suggests that resistance could rise again in this region if refuges are not sufficiently abundant. The similar resistance of pink bollworm to Cry1Ac in lab- and field-selected strains from China, India and the U.S. provides a basis for developing resistance management practices that extends beyond national borders.

## Materials and Methods

### Insects and toxins

We used three strains of pink bollworm: JL46 from Jianli, Hubei Province of China; and AZP-R and APHIS-S from Arizona in the U.S. APHIS-S is a susceptible strain reared in the laboratory in Arizona and then China without exposure to Bt toxins^35,46,47^. AZP-R is a Cry1Ac-resistant strain that originated from pooled survivors from 10 populations derived in 1997 from Arizona cotton fields^48^. As part of an F_1_ screen used for Cry1Ac resistance monitoring in 2013, the JL46 strain was created by a single-pair cross between a field-collected male (#46) from Jianli and a resistant female (genotype *r1r1*) from AZP-R. From the F_1_ progeny of that cross, we selected for survivors on 10 μg Cry1Ac per mL diet and used DNA-based screening from F_2_ single pair crosses to eliminate individuals harboring the *r1* cadherin allele (Fig. S1). The end result was the homozygous *r15r15* JL46 strain.

All three strains were maintained at 29 ± 1 °C, 50 ± 10% relative humidity (RH) and a photoperiod of 16:8 (L:D) as previously described^47^. Larvae were reared on wheat germ diet^46^ and AZP-R and JL46 were selected every fifth generation on 10 μg Cry1Ac protoxin per mL diet (n = 960 larvae per strain per selection). For all exposure of larvae to toxins in diet for bioassays and selection, we used the protoxin form of Cry1Ac and Cry2Ab purchased from Zhongbao Biotechnology Company, Beijing, China. For cell assays, we used activated Cry1Ac toxin purchased from Marianne Pusztai-Carey (Case Western Reserve University, Cleveland, OH), as described previously^35^.

### *PgCad1* cloning

We cloned and sequenced full-length complementary DNA (cDNA) and partial genomic DNA (gDNA) of *PgCad1* from fourth instar larvae of JL46 strain survivors on a diagnostic concentration of Cry1Ac (e.g. 10 μg Cry1Ac protoxin per mL diet). Total RNA and gDNA were extracted from fourth instar individual larvae (n = 8) using TaKaRa MiniBEST Universal RNA Extraction Kit (TaKaRa). M-MLV Reverse Transcriptase (Promega) was used for first strand cDNA synthesis and full-length *PgCad1* cDNA was PCR amplified using primers F1 + R1 and F2 + R2 (Table S1) Primers were designed based on full-length cDNA sequence of *BtR-s* allele (Genbank Accession Number AY198374.1) from APHIS-S. For cloning gDNA flanking the *r15* mutation site, we used gF46 + gR46 (Table S1) and LA-Taq (TAKARA, Dalian, China) to PCR amplify the PgCad1 partial gDNA fragment. PCR conditions were: 94 °C for 2 min, followed by 32 cycles at 98 °C for 10 s, 60 °C for 30 s, and 72 °C for 3 min, and a final extension at 72 °C for 7 min. PCR products were cloned into pGEM®-T Easy Cloning Vector (Promega) cloning vector and DNA sequencing was performed as previously described^35^.

### Bioassays

Diet bioassays were conducted to compare larval susceptibility of JL46 and APHIS-S strains to both Cry1Ac and Cry2Ab. The concentrations of Cry1Ac (in μg Cry1Ac protoxin per ml diet) were 0 (control), 0.05, 0.1, 0.15, 0.2, 0.25 and 10 for APHIS-S (n = 120 larvae per treatment); 0 (control), 1.25, 2.5, 5, 10, 20 and 40 for JL46 (n = 72 larvae per treatment); and 0 (control), 0.125, 0.25, 0.5, 1, 2 and 4 for the F_1_ offspring from both APHIS-S × JL46 reciprocal crosses (n = 96 larvae per treatment). The concentrations of Cry2Ab (in μg Cry2Ab per ml diet) were 0 (control), 0.03, 0.06, 0.12, 0.24, 0.48, 0.96 and 1.92 for APHIS-S (n = 72 larvae per treatment); and 0 (control), 0.05, 0.1, 0.2, 0.4, 0.8 and 1.6 for JL46 (n = 72 larvae per treatment) for JL46.

Boll bioassays were conducted to observe specific life history traits for JL46 and APHIS-S reared from larvae on either non-Bt cotton bolls (Simian-3) or bolls from Cry1Ac Bt cotton (GuoXin H318). We tested 10–12 Bt cotton bolls and 13–15 non-Bt cotton bolls in three replicated tests for each strain (n = 300 per boll type per replicate). Neonates were transferred to bolls using a fine brush and we counted and recorded the number of entry holes, exit holes, the date of pupation, pupal weight, and sex ratio as described previously^35^.

### Inheritance of resistance to Cry1Ac

We set up two reciprocal mass crosses including 20 female APHIS-S × 20 male JL46 and 20 female JL46 × 20 male APHIS-S in 2 L cylindrical boxes. Adults were allowed to mate and lay eggs. Newly emerged F_1_ neonates were tested in bioassays using the Cry1Ac concentration series outlined above for reciprocal crosses. Neonates from JL46 and APHIS-S were included as reference strains. Dominance (*h*), which varies from 0 for completely recessive resistance to 1 for completely dominant resistance, was determined from survival at 10 μg Cry1Ac per mL diet adjusted for control mortality.

### Genetic linkage between resistance to Cry1Ac and *r15*

To test for genetic linkage between resistance to Cry1Ac and *r15*, we generated F_1_ progeny from a single-pair cross between a single APHIS-S male and one JL46 female. Then, five F_1_ males were paired with JL46 resistant females to generate the backcross families. The F_2_ progeny from these five families were bioassayed on diet with or without 10 μg Cry1Ac per mL diet to determine whether resistance to Cry1Ac is tightly linked with *PgCad1 r15*. Because crossing over only occurs in male Lepidoptera^49^, we only used F_1_ males to produce backcross families and test the tightness of genetic linkage. A total of 90 neonates were bioassayed for each backcross family, including control diet (n ~ 30) and on 10 μg Cry1Ac per mL diet (n ~ 60). We conducted the allele-specific PCR test according to method mentioned above to identify the genotype of fourth instar larvae either from control diet or from treated diet for each backcross family. In total, 258 larvae were genotyped, including 153 on control diet (n = 30, 32, 30, 30 and 31 for each backcross family) and 105 larvae on 10 μg Cry1Ac per mL diet (n = 20, 24, 20, 20 and 21 larvae for each family).

### Expression vectors and transfection of cultured insect cells

Total RNA isolated from 4th instar larval midguts from JL46 and APHIS-S was used to make cDNA as indicated above. Amplicons corresponding to full-length *r15A* and *r15B* were PCR amplified from JL46 cDNA using high-fidelity thermostable DNA polymerase (Thermo) (see Table S1 for primers). The full-length *PgCad1_s* was amplified from APHIS-S cDNA. Each cDNA was cloned into the pIE2-EGFP-N1 expression vector containing the green fluorescent protein (GFP) reporter gene^50^ used to generate the fusion proteins r15APgCad1-GFP, r15BPgCad1-GFP, and sPgCad1-GFP. All *PgCad1* PCR products were amplified to include *Eco*RI and *Sac*II restriction sites for cloning into pIE2-EGFP-N1. Recombinant vectors were used to transfect Hi5 cells (BTI-Tn-5B1–4 cell line provided by Peter Tijssen, University of Quebec, Canada). The endoplasmic reticulum (ER) of insect Hi5 cells was labeled by pDsRed2-ER as previously described^45^. Transfection and calculation of transfection efficiency were performed as previously described^35^.

### Expression of recombinant PgCad1-GFP in Hi5 cells

Equal amounts (2 μg) of pIE2-sPgCad1-GFP, pIE2-r15APgCad1-GFP, pIE2-r15BPgCad1-GFP or the empty vector pIE2-GFP (as a negative control) were used to transfect Hi5 cells. Cells were seeded, recovered, lysed and finally analyzed by immunoblotting. After cell lysis, the total protein concentration for each sample was determined by using Pierce® BCA protein assay kit (Thermo) following the manufacturer’s instructions and an equal amount of total protein (40 μg) was separated by SDS-PAGE, transferred to a polyvinylidene difluoride membrane, and successively incubated with primary and secondary antibodies as previously demonstrated^35^.

### Cell toxicity assays

Twenty-four hours post-transfection, we observed cytotoxicity of the cells with and without Cry1Ac using fluorescence microscopy (Nikon). Whereas pIE2-sPgCad1-GFP-transfected cells were treated either with no toxin or with 10 μg per mL Cry1Ac, pIE2-r15APgCad1-GFP and pIE2-r15BPgCad1-GFP and the empty vector pIE2-GFP were either treated with no toxin or with 40 μg per mL Cry1Ac. Toxicity was estimated by the proportion of swollen cells as previously shown^50^. Each treatment was repeated three times with a total of six fields of vision used to quantify cell mortality.

### Statistical analysis

We analyzed larval diet bioassay data with probit regression using IBM SPSS Statistics 22.0 to determine LC_50_ values and their 95% fiducial limits (FL), and slopes of the concentration-mortality lines and their standard errors (SE)^51^. We also used probit regression to analyze cell toxicity data to determine the concentration of Cry1Ac causing swelling of 50% of cells (EC_50_) and its 95% FL. We estimated the dominance parameter *h* based on survival at the diagnostic concentration adjusted for control mortality as described previously^52^. In the linkage analysis, for the backcross progeny on untreated diet, we used a one-sample t-test to determine if the observed percentage of larvae that were *r15r15* differed significantly from the 50% expected under random segregation. We used Fisher’s exact test to determine if the proportion of larvae that were *r15r15* differed significantly between the survivors on treated and untreated diet. For the boll bioassays, we calculated larval survival in each of the three replicates as the number of survivors divided by the number of entry holes. We also calculated relative survival for each of the three replicates as larval survival on Bt cotton divided by larval survival on non-Bt cotton. We used standard t-tests to determine if significant differences occurred between the resistant strain (JL46) and the susceptible strain (APHIS-S) in larval survival on Bt cotton and relative survival, as well as in larval survival, development time, and pupal weight on non-Bt cotton. In addition, we used standard t-tests to determine if significant differences occurred for JL46 between Bt and non-Bt cotton in development time, pupal weight, eggs laid per female, and hatching rate of eggs. For JL46, we also used Fisher’s exact test to determine if survival from neonate to adult and the proportion of adults that were female differed significantly between Bt and non-Bt cotton.

## Supplementary information

Supplementary Figures S1–8

Supplementary Tables S1–5
