# Supplementary material for: Transposon insertion causes cadherin mis-splicing and confers resistance to Bt cotton in pink bollworm from China
Source: Sci Rep. 2019 May 16;9:7479. doi: 10.1038/s41598-019-43889-x (PMC6522560; doi:10.1038/s41598-019-43889-x)
Supplement: Supplementary file 1 — Supplementary Figures S1–8 [file 41598_2019_43889_MOESM1_ESM.pdf]

Transposon insertion causes cadherin mis-splicing and confers resistance to Bt cotton in pink bollworm from China

Supplementary Figures S1-S8

Ling Wang[1,2], Jintao Wang[1,3], Yuemin Ma[4], Peng Wan[1], Kaiyu Liu[4], Shengbo Cong[1], Yutao Xiao[5], Dong Xu[1], Kongming Wu[2*], Jeffrey A. Fabrick[6], Xianchun Li[7] & Bruce E. Tabashnik[7]

[1]Key Laboratory of Integrated Pest Management On Crops in Central China, Ministry of Agriculture, Hubei Key Laboratory of Crop Disease, Insect Pests and Weeds Control, Institute of Plant Protection and Soil Fertility, Hubei Academy of Agricultural Sciences, Wuhan 430064, China. [2]State Key Laboratory for Biology of Plant Diseases and Insect Pests, Institute of Plant Protection, Chinese Academy of Agricultural Sciences, Beijing 100193, China. [3]Hubei Insect Resources Utilization and Sustainable Pest Management Key Laboratory, Huazhong Agricultural University, Wuhan 430070, China. [4]School of Life Science, Central China Normal University, Wuhan, 430079, China. [5]Agricultural Genomics Institute at Shenzhen, Chinese Academy of Agricultural Sciences, Shenzhen, 518120, China. [6]USDA, ARS, U.S. Arid Land Agricultural Research Center, Maricopa, Arizona 85138, USA. [7]Department of Entomology, University of Arizona, Tucson, Arizona 85721, USA.

* Correspondence and requests for materials should be addressed to K.W. (E-mail address: kmwu@ippcaas.cn)

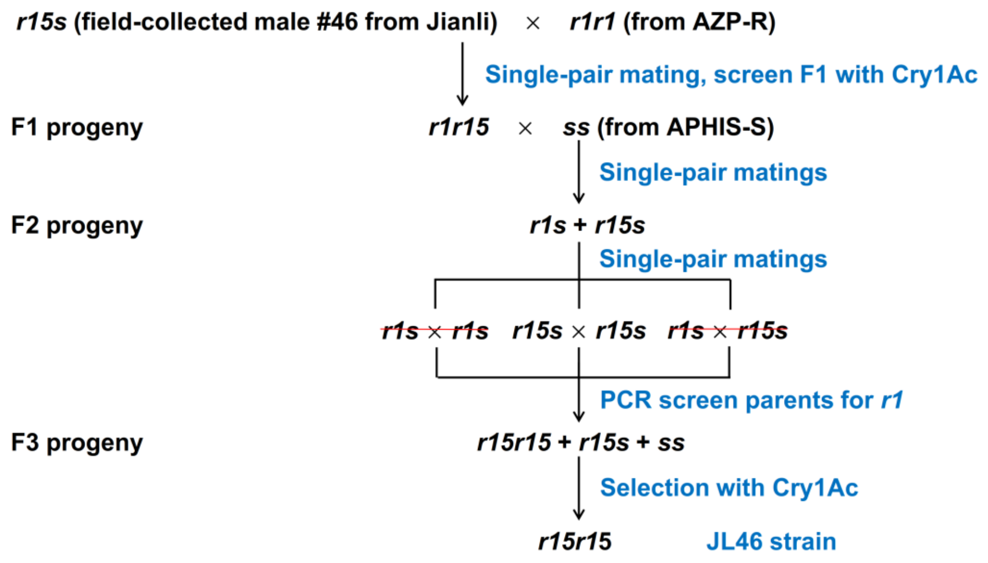

Figure S1. Isolation of pink bollworm resistant strain JL46. Pink bollworm strain JL46 was started from a single-pair cross between a field-collected male (#46) from Jianli in Hubei province of the Yangtze River Valley and a female from a Cry1Ac-resistant strain AZP-R (cadherin genotype *r1r1*) from Arizona. Their F_1 progeny (family #46) were screened with 10 μg Cry1Ac protoxin per ml diet, a diagnostic concentration of Cry1Ac. Survival of the F_1 progeny at the diagnostic concentration was 47% and recessive resistance to Cry1Ac of pink bollworm was described before[1,2], indicating that the field-collected male parent of family #46 carried only one recessive allele at the cadherin locus. Sequencing of cDNA from the resistant F_1 progeny showed that male #46 had two transcripts of cadherin allele, which we name *r15A* (Figures 1, S2 and S3) and *r15B* (Genbank accession numbers KY814704 and KY814705, respectively).

Survivors from family #46 were reared to adults and single-pair crosses between these survivors and adults of the opposite sex from the susceptible strain APHIS-S (cadherin genotype *ss*) from Arizona were conducted separately to produce resistant strain JL46. We used PCR to identify *r1* allele for the parents of all single-pair F_2 families[3], and retained only the families from pairs of parents without carrying *r1* allele (*r15s*), then these single pairs of these F_2 progeny were selected by exposing larvae to diet containing the diagnostic concentration of Cry1Ac. The individuals survived on the diagnostic concentration were homozygotes *r15r15*, which were reared their progeny as strain JL46, and feeding larvae of JL46 with diet containing the diagnostic concentration of Cry1Ac every fifth generation to maintain the resistance to Cry1Ac.

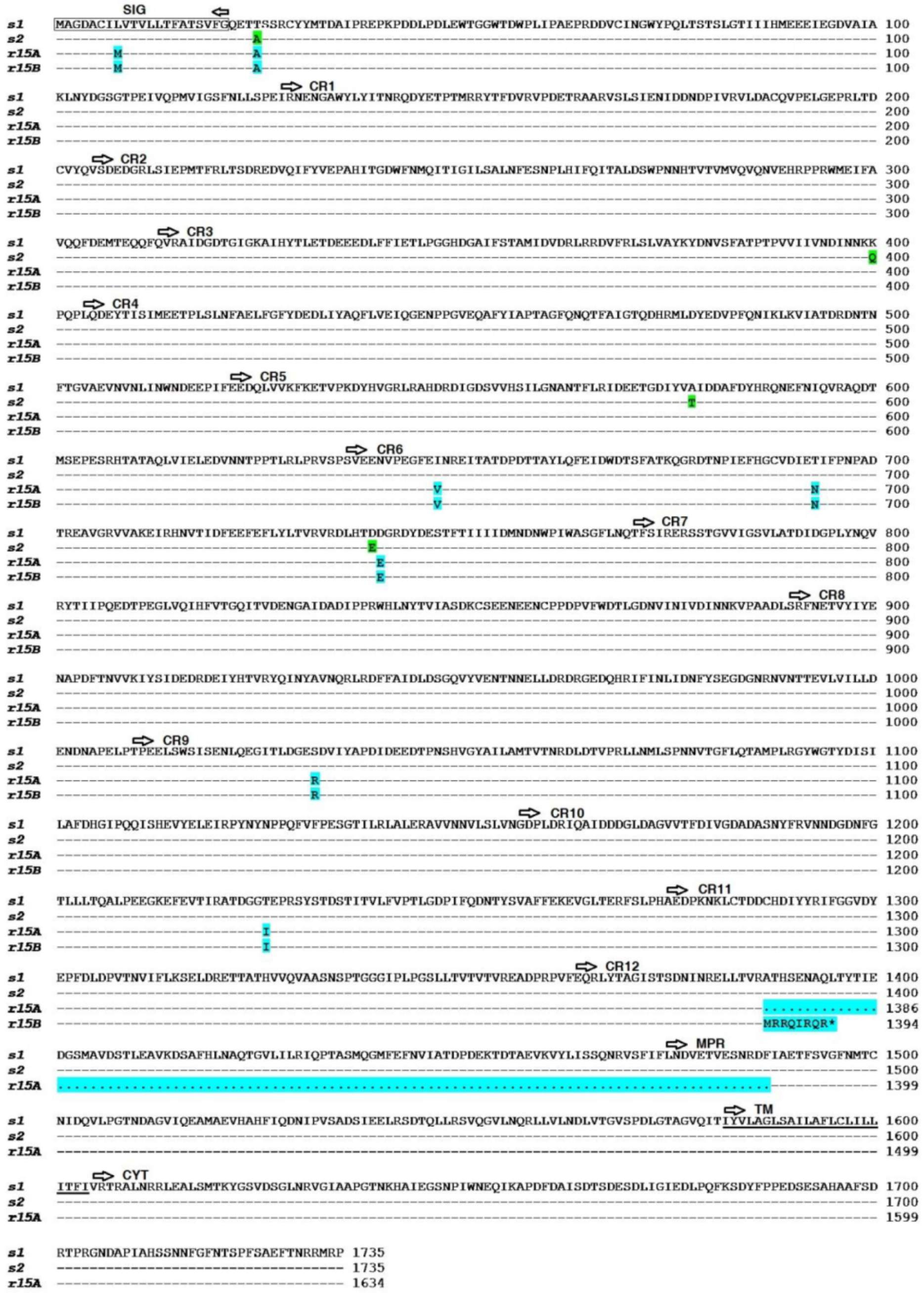

Figure S2. Predicted amino acid sequence of pink bollworm cadherin protein PgCad1 for alleles *s1* (GenBank accession no. AY198374) and *s2* (GenBank accession no. MF276974) from susceptible strain APHIS-S; and *r15A* (GenBank accession no. KY814704) and *r15B* (GenBank accession no. KY814705) from resistant strain JL46). The *s1* allele was sequenced in 2003 from APHIS-S reared in Arizona[1] and *s2* was sequenced in 2015 from the subset of APHIS-S reared in China[4]. Shown are the signal sequence (SIG), cadherin repeats (CR1-CR12), membrane-proximal region (MPR), transmembrane region (TM) and cytoplasmic region (CYT). The blue shading indicates differences in *r15* compared with *s1*: deletion of 101 amino acids (1387 to 1487 for r15A, indicated by dots) and frameshift mutation for r15B and seven single amino acid substitutions (L8M, T25A, I647V, T693N, D740E, S1032R, T1226I). The green shading shows differences in *s2* versus *s1*: four single amino acid substitutions.

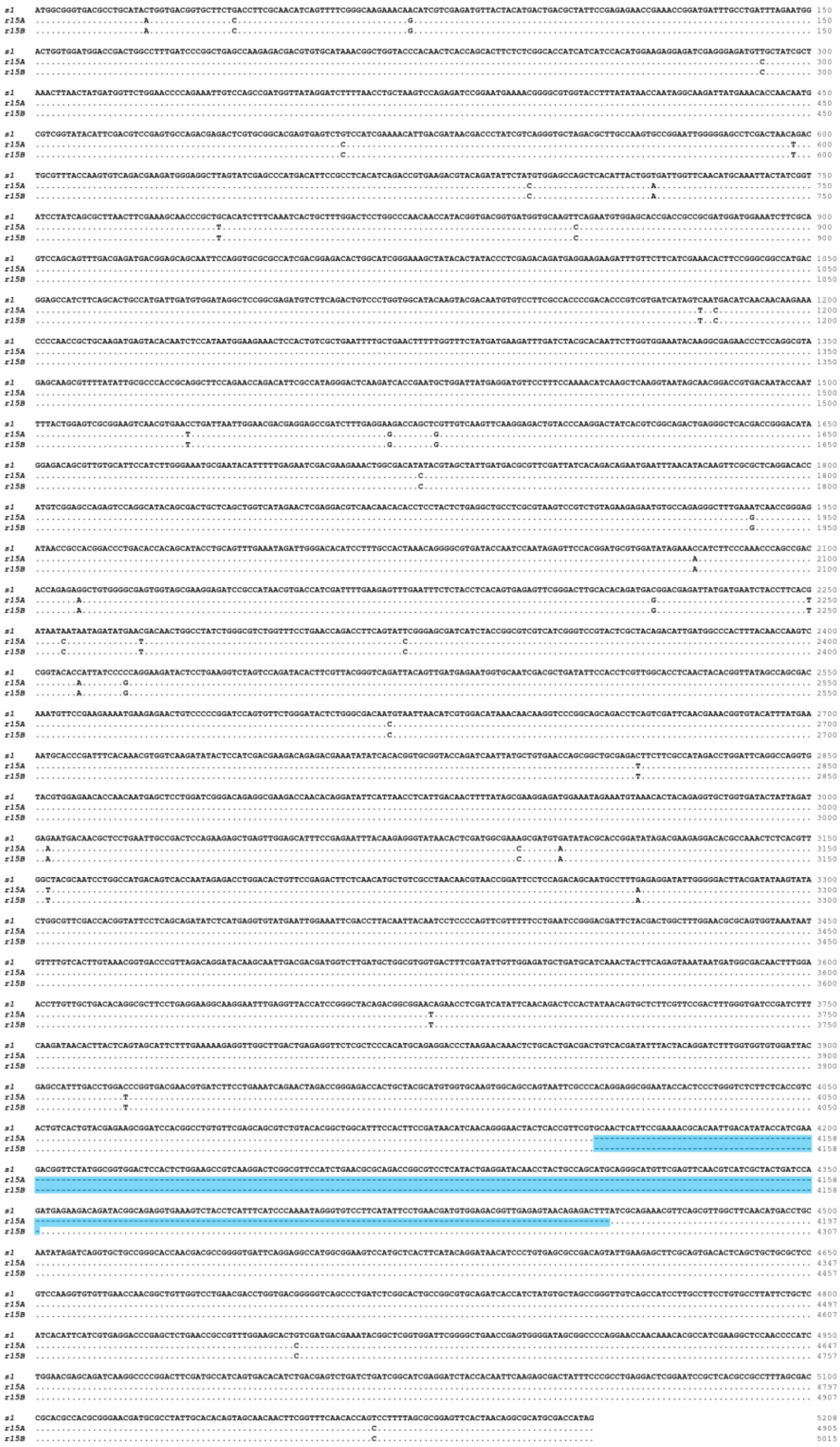

Figure S3. The full length cDNA sequence of pink bollworm cadherin protein PgCad1 for alleles *s1* (GenBank accession no. AY198374) from susceptible strain APHIS-S; and *r15A* (GenBank accession no. KY814704) and *r15B* (GenBank accession no. KY814705) from resistant strain JL46). The blue shading indicates differences in *r15* compared with *s1*: deletion of 303 bp for *r15A* (4159 to 4461, indicated by hyphens) and 193 bp for *r15B* (4159 to 4351, indicated by hyphens).

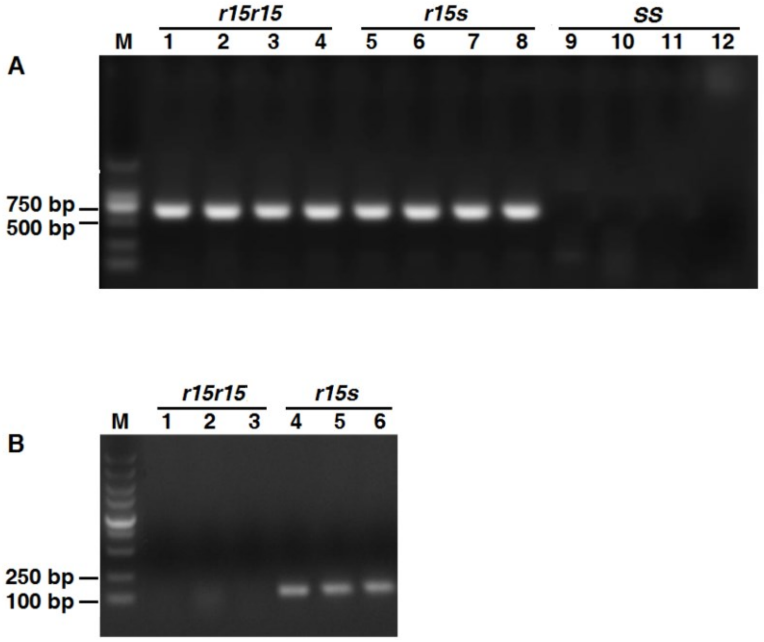

Figure S4. PCR detection of *PgCad1* genotype using primers in Table S1. (A) Primers for *r15* (*r15*F and *r15all*R1) generate a single band of 652 bp in *r15r15* from JL46 (lanes 1-4) and in *r15s* (F_1 progeny of APHIS-S × JL46; lanes 5-8); and no band in *ss* from APHIS-S (lanes 9-12). (B) Primers for *s* (not*r15*F and *r15all*R2) generate no band from *r15r15*(lanes 1-3) and a single band of 152 bp from *r15s* (lanes 4-6).

```
CAGGGAACTACTCACCGTTCGTGTAAGTTCTCCTTCTCTCTCTCTATTTAAGAGCTGCGCTCTTGTCGGTGGAGTAATCGCCATTCCTCTCTTCTTCCCGCCAAAACCTTCACCTCCCGA 120
TACGACACGACCTGCACCTTCTCTTTTATTTGTTTCATAAATGTTATCCTAGGTCTAGGTCCCTTCCTCTCTTCCCTTAAATTTTTTCTTCTATGATGTTTGTTATAAAAGAATCGTGTC 240
GTATCAGGTGGCCAATCATATTTCCTCTCCGGTTCTCTATAGTATCTTCAACTCTTTCCAGTACTTTTTCATTTGACACCATTTCCGTCCAGCTTTTTACTTATTAATGATATTAATAAA 360
TAAAAAAACCCCTTCTCTACCCCTTCCTCTCTTCCCTTCAATTTTTCCTTCTATAATGTTTGTTATAAATGAATCGTGTCGTATCAAGTTCTACTTATTTGTACCATATTCATTAGCTCC 480
GACCACATTTCACAGGGGCATTAACTACCAAAGAATGAATGAATTAAGAATGAGTATTTATTCGCATAATCAATTACAAGAATTGTGTTCGAAATACCGAAACAGACAGAATAGTAAAAA 600
ATAAGTTTACCAAAGCAATCATCAATTATTGAAAATAAATGAATTCAAGATATAAAAAATTGTTGATTTGGATAAAAAATAATAAGAATAAAAGGACACTAAAAAGGAAATAAAAGTCTA 720
CAAGTAAATGTTATACTTACCTACCTATCACTTTTATTGTAAGCCCTCATCATCATCAATTTAAGAGCCACGCTCTTGTCGGTGTAGCATTTTCCATTCCAGTCTATCAAAGGCCAATTC 840
CTTGACTTCCCTATAAGACACGACGTTAACATTTTCTTTAATCTGTTCCATGTAAGCTCTTCTTGGTCTTCCCCTTTCTCCCTTTCCTTCTAGCTTTCCTTCTATGATGTTTTTAATAAA 960
TTCGTCGTGTCAAAATTGTAAGCCCTATGCTATAGTTTTTTGTCTTTAGTAGGTATTTGTGTCTCATCACCTTTTTGTCTGATCGCATTTACCACTTTAATTTAAGCTTTTTATAAATTC 1080
CCTACCAACGACTGTTCCTTCAAATTCACTTACAAACCGATTCTTTGATCAGGCAACTCATTCCGAAAACGCACAATTGACATATGGGCTATTATTTTATTTTGGGTCCCATAACCTGTC 1200
TCTCACGTTCAAACACGATTTTTATAAAGCAAACAATTATTTTTTAAATAATTACACTATTGGTACATTACTGATGTTTTATACTCGTGCTTTATATTCCACGTTTTTATCAAAATTATT 1320
TGAATTTAACCAAAAACACGGTAACTAAGTTGTACTTTTGAGTAACGCAGTTGTTTCCCTACAAAATTCCACTGTCCCTCTCATATATTTACTCCATATAAACACACATTACTTAAAAAC 1440
TCTTCAAGACGCTATTAAAATGTAATATTTGCGGTAATAACACGTAATTTAATCTTTACTTTAACAATAATAAATATTATTTTCCTTTATCAGTTTTTAATTATTCCAAAATATTTATTG 1560
TGTCCCCGCGGCAAACCGAAAAATTACGAACACTATGTTACCATACGAAACTGAGTCTTAAAATTAGAATTTTGTATGCATTTGCGCCAAAAGTCGTATAACATATAATCAAATATTATT 1680
GGGGCGCGGGGGGAGTGCGTGGCTTTGAAATTCGCCGAGTCAGTCGCGAGGACGCCGGCAGGATAGGTGCGTGTATTAATTGGGAGACCGCTGAGTGACCGCTGTGATAGGTGAGTTTTT 1800
TGAATATTGAATAATCTTTTACGTTATATCTTTAAAAAAAGGAGCAAATATTTATTGTACACGATCTGTTTTATAAAATGAATTACCATATGAGTGTTATTTTAGAATACACGTGGTTTC 1920
TACAACTGAGTTACCGATGCACTGCGTTACTGCGAAATGTAACGCAGTTGTAGCCATCGTAACGCAGTGGAAATACTCAAAGATTTTGCTCAATATTTACAAATTTAATAGTTAAATAAT 2040
AGTTTAATTTATAATTATTTCGTTACAGAGTGTCGAGTTTATCGTCCACAAGTCCCTAGTCAACAACGTGTTGACCATCGGTAGTGTTTCGAGCAGGGTAGCGTGCCTGATACTCAGAAT 2160
ATCATCTCGATATTCGCTGACTGTCATCCAGGTATACGCTCCGACCTCGGGACACCACGATGACGAAGTGGAGACTATGTATGTGGAGATTTCTAAAGCCATGCATAGCCATAAAAGCCA 2280
CTACACGATTGTGATGGGGGACTTTAATGCACAGTTGGGGAAGCGTTGCGGTAACGAGCTGAGAGTAGGGCAATTTGGATTTGGGGTTTGGAACACCAGAGGCCGGATGCTGGCGGACTT 2400
TATGGAGAAGAAGAACTTCTATATGATGAACTCCTTCTTCAGAAAGCCCGCACCCGCAAATGGACCTGAATAAGTCCCAGTGGAAATTATAAAAACGAGATCGATTTTATCATGTCGACG 2520
AAAAAGCACATATTCAATGATGTCTCTGTGATCAATGCCGTTAAGACGGAAAGCGAACGAACGGGTTTCCGAAAAGGCTTTAGCACCATAGACCACATCCATACGATGCGGCAGGTTATA 2640
CAGAAGACCGAAGAGTATAATCTGCCACTTTGCTTGGCGTTTGTGGACTATGAGAAAGCCTTTGATTCGATCGAGACCTGGGCGGTGTTGCAGTCTCTTCAGAGGTGCCAAGTGGACCAT 2760
AGGTACATCGAAGTGCTAAGGGACCTCTACCAAAATGACACTATGTCAGTTCGAGAACAGAACCAGAGCTCTAATCCGATCCAACTGCAGCGAGGAGTGAGACAGGGAGATGGTATCTCT 2880
CCGAAACTGTTCACTGCTGCATTGGAGGATATTTTTAAGCTTCTGGACTGGAAAGGATTTGGCATCAACATCAACGGCGAGTACCTGACGCACCTTCGATTTGCCGATGATATAGTCCTA 3000
ATGGCTGAGACCCTGGAAGACCTGAACACCATGCTCGAGCATCTCAGTAACGCATCCCAATGAGTGGGTCTTAGCACGAACATGGCAAAGACAAAAATTATGTCCAATGACCATGTCGCA 3120
CCCACTCCAGTTCAGGTTGGGAACGTTACACTCGAAGTTGTAGACCAGTATATTTACCTAGGACAAATAATCCAGTTAGGTAAGTCCAACTTCGAGAAAGAGATCTCTCGTCGGATCC AA 3240
CTCGGATGGGCAGCGTTCGGGAAGCTGCGGAATATCTTCTCGTCCAAAATACCTCAGTGTCTCAAGAGGAAAGTCTTTGACCAGTGCGTGTTGCCAGTGATGATCTACGGCAGTGAGACG 3360
TGGCCGCTTACTATGGGTCTCGTGAGGAGGCTCGGTGTCGCTCAGCGGGCAATGGAGAGAGCTATGCTCGGTATTTCCCTGCTCGATCAAATCAGAAATGAGGAGATCCGCAGGAGAACT 3480
AAAGTCACCGACATAGCTCGGCAAATTGCAAAGCTGAAGTGGCAGTGGGCAGGACACATAGCGAGGAGAACCGGTGAAGGTCGCGGAAAGCCGCTGGATGCGGGCAGCGCAGGACCGATC 3600
GTCGTGGAGATCCTTGGGGGAGGCTATGCCCAGCAGTGGGCGTCGTAAGGCTG(ATGACGATGATGACATACATACATACA)TAAACTCATGCCTATTTCCCACCGGGGTAAGCAGAGACTA 3720
TAGAATTCCATTTGCTGAAAACGAATTGAATGAATTTGATGTCCCATTAAATATATTTGACTCAGACAGAAACTAATGTATGATTAATATTGTTTTTTTGAGAAGTTTTGATTTTATTTT 3840
ATTCTAAGAGAGTTTTGTTTAATTTGTTATGGTTTCAATTTAATTGTATTCTGTTTTTAATTGTTTTCAGTAAGTTAAACACCATAGATGCGGAAAGT(TTACCATTATGTTACTTAC)TAA 3960
ACTACTGCGTTACTTTTTTGTCCCCCATTTTTATGGAGACATTTTCAGACTTAGAACTAAAACTACCTAAGTATATTTTAAAATTTTTGACTTTTATTGATTATTTTTAATATATAAATG 4080
ACCTCCATAAATAAAATGCTTGAACTGCTATTGTTTTCTATTTTATTAAAAAAGTTTATCTCATAAAATCAAAATTAGGTAACGAAGTGGTAAACCTAACCATAAAAAGATGTATTA GAC 4200
TGAAATGTTTCGTGATTTAATAATATAAGGTCAAAAATCTTTTATCTTAATTTAAAAAAGAAAAGGTTTGAATTTAATGTGAACGTTCCCAATAATAAAACTACATAATGTAACAAAGTA 4320
ACACAGTTGTTGTTTTTCTGCACCCTCCCTAGTATAAATATAAAAATAAGTGAAAAATAATTGGTAAACATCGCTGCAAATTCCTGTAAAATGTAATTTAAATATTATTATTCTTATAAT 4440
AGTAAGAAATATTAACAAACAATAGCATAAATAAAAAAAATTTTTTTTGGCGAATAAGTTAACCATGGCACCCAGAATAAAATAATAGCCCATATACCATCGAAGACGGTTCTATGGCGG 4560
TGGACTCCACTCTGGAAGCCGTCAAGGACTCGGCGTTCCATCTGAACGCGCAGACCGGCGTCCTCATACTGAGGATACAACCTACTGCCAGCATGCAGGGCATGTTCGAGTTCAACGTCA 4680
TCGCTACTGATCCAGGTAATGGGGCACAGCTACGCAAGGACTTTGAGGCAAGGGGGTAACAGTGTTGTCTGAAAAAAAGGCGGTGATCGGCGTCCTCACACTGAGGATACAGTCACAGGA 4800
TTTGTGTTCCACGTCATCAGCTGATTCAGAGAGGCGTGCGCTATAGAATCATAACATAAACATAAACAGCCTATATACGTCCCACTGCTGGGCACAGGCCTCCCCTCAATCAACCGGAGG 4920
GGTATGGAGCATACTCCACGCTACTCCAATGCGGGTTGGTGGAGGTGTTTTTACAGTTTGTAGAACGCTTGCCTCTCACTTTGAGGTCGCAAGTTCGAATCCAGCACAGGCCTAAACCAA 5040
TTATTGTCGAATTTGTTTTCGAATTTATGTTTGGATCATAAATGATTATCACGTGCTCAACAAAAACATCGTGAGGAAACCCGAGAAATGCATTTCCGAAGGTATGTGACCTAACCTGTA 5160
TTGGGCTGGTTTTCCCTGGTTGGAAGGTAAGACATGCCTGTCTTACCTTTCGCTTCTTTAAAAACCGGACTTGTCAAATCTTCAGGTTAGGCAAGCGGACCCTGTGAAATACGGATTAAG 5280
GCTAGGGAGATGATGATGATGAGTCACTTCCAAGTTTTTAACCAAAAGGATTGGATTCGGATTCGTAAATGTAAATATTAGACCTCTGTAACATAATAGCCACATAGCTAGGTGATCAAG 5400
GAACCAAAGACAATACTTACTACCTTATTCAAGATTAATTTCCTGAAGAATGTAAAATTTCCAAACACAAAAATCAGTGAGTACTTATTTTATATTCTTACTTATTCCAG ATGAGAAGAC 5520
AGATACGGCAGAGGTGAAAGTCTACCTCATTTCATCCCAAAATAGGGTGTCCTTCATATTCCTGAACGATGTGGAGACGGT 5601
```

Figure S5. Sequences of the three nested TEs inserted into Exon 28 of r15 gDNA sequence. Blue=MITE-1_PGo, Green=RTE-5_PGo, Gray=SINE-1_PGo. TSD= italic and underlined letters. TIR=underlined letters. Microsatellite repeats of (AYG)4(ACAT)4 for RTE-5_PGo and of (TTAY)4 for SINE-1_PGo are indicated with a pair of parentheses.

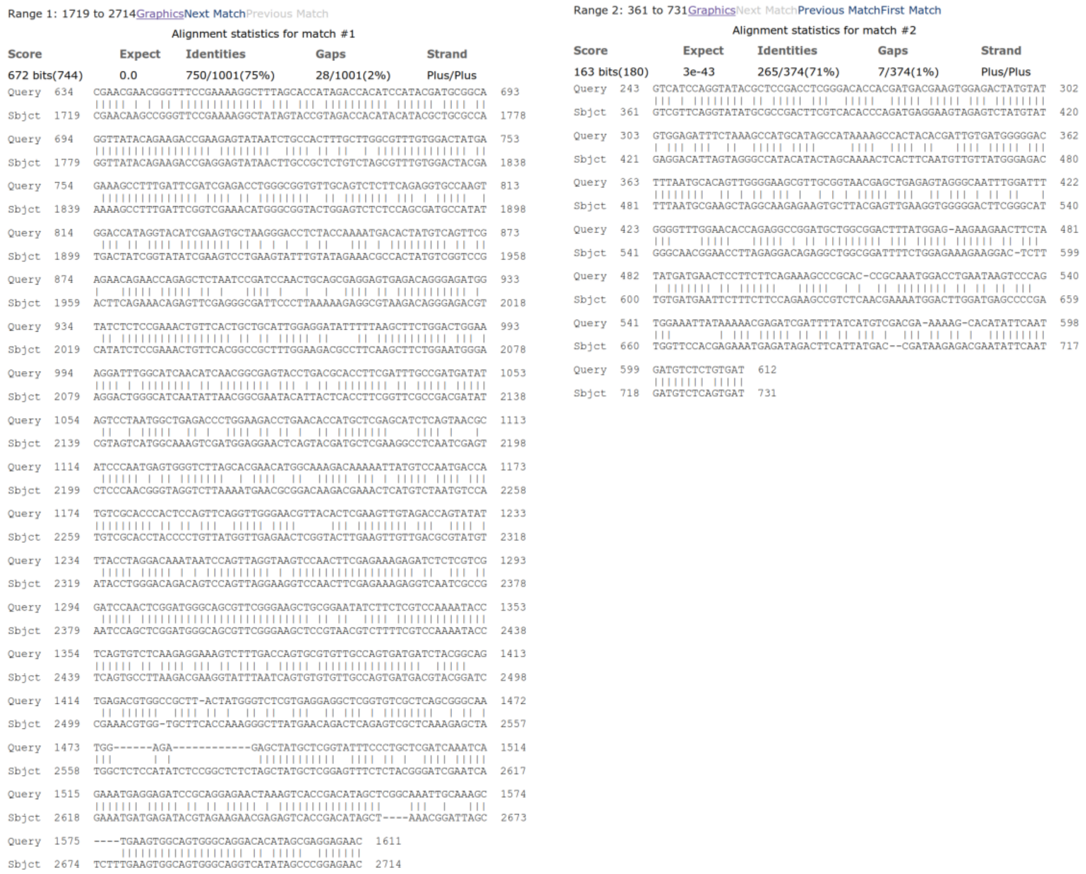

Figure S6. Nucleotide sequence alignment between RTE-5_PGo and RTE-5_DPl. Query=RTE-5_PGo, Subjct = RTE-5_DPl.

Range 1: 436 to 770GraphicsNext MatchPrevious Match

Alignment statistics for match #1

Score	Expect	Method	Identities	Positives	Gaps
516 bits(1328)	0.0	Compositional matrix adjust.	249/336(74%)	287/336(85%)	14/336(4%)

```
Query  100  ERTGFRKGFSTIDHIHTMRQVIQKTEEYNLPLCLAFVDYEKAFDSIETWAVLQSLQRCQV  159
            E+ GFRKG+ST+DHIHT+RQVIQKTEEYNLPLCLAFVDYEKAFDS+ETWAVL+SLQRC +
Sbjct  436  EQAGFRKGYSTVDHIHTLRQVIQKTEEYNLPLCLAFVDYEKAFDSVETWAVLESLQRCHI  495

Query  160  DHRYIEVLRDLYQNDTMSVREQNQSSNPIQLQRGVRQGDGISPKLFTAALEDIFKLLDWK  219
            D+RYIEVL+ LY+N TMSVR Q QSS  I L+RGVRQGD ISPKLFTAALED FKLL+W+
Sbjct  496  DYRYIEVLKYLYRNATMSVRLQKQSSRAIPLKRGVRQGDVISPKLFTAALEDAFKLLEWE  555

Query  220  GFGININGEYLTHLRFADDIVLMAETLEDLNTMLEHLSNASQMA-------KTKIMSNDH  272
            G GININGEY+THLRFADDIV+MA+++E+L+TMLE L+  SQ         KTK+MSN H
Sbjct  556  GLGININGEYITHLRFADDIVVMAKSMEELSTMLEGLNRVSQRVGLKMNADKTKLMSNVH  615

Query  273  VAPTPVQVGNVTLEVVDQYIYLGQIIQLGKSNFEKEISRRIQLGWAAFGKLRNIFSSKIP  332
            VAPTPV V N  LEVVD Y+YLGQ +QLG+SNFEKE++RRIQLGWAAFGKLRN+FSSKIP
Sbjct  616  VAPTPVMVENSVLEVVDAYVYLGQTVQLGRSNFEKEVNRRIQLGWAAFGKLRNVFSSKIP  675

Query  333  QCLKRKVFDQCVLPVMIYGSETWPLTMGLVRRLGVAQRAM------ERAMLGISLLDQIR  386
            QCLK KVF+QCVLPVM YGSETW  T GL+ RL VAQRAM        AMLG+SL D+IR
Sbjct  676  QCLKTKVFNQCVLPVMTYGSETWCFTKGLMNRLRVAQRAMALHISGSLAMLGVSLRDRIR  735

Query  387  NEEIRRRTKVTDIARQIAKLKWQWAGHIARRTGEGR  422
            N+EIRRRT+VTDIA++I+ LKWQWAGHIARRT +GR
Sbjct  736  NDEIRRRTRVTDIAKRISSLKWQWAGHIARRT-DGR  770
```

Range 2: 1 to 114GraphicsNext MatchPrevious MatchFirst Match

Alignment statistics for match #2

Score	Expect	Method	Identities	Positives	Gaps
114 bits(285)	3e-31	Compositional matrix adjust.	57/114(50%)	78/114(68%)	14/114(12%)

```
Query  1    MYVEISKAMHSHKSHYTIVMGDFNAQLGKRCGNELRVGQFGFGVWNTRGRMLADFMEKKN  60
            MY +IS+A+H+ K+H+ +VMGDFNA+LGKR   EL+VG FG G  N RG+ LADF+EK+
Sbjct  1    MYEDISRAIHTSKTHFNVVMGDFNAKLGKRSAYELKVGDFGHGQRNLRGQRLADFLEKEG  60

Query  61   FYMMNSFFRKPAPAN----GPE----------MSTKKHIFNDVSVINAVKTESE  100
             ++MNSFF+KP+        P+          M+ K+ IFNDVSVI+ VKT S+
Sbjct  61   LFVMNSFFQKPSQRKWTWMSPDGSTRNEIDFIMTDKRRIFNDVSVIHRVKTGSD  114
```

Figure S7. Amino acid sequence alignment between RTE-5_PGo and RTE-5_DPl. Query=RTE-5_PGo, Subjct = RT-5_DPl. Range 2 is the EP domain. Range 1 is the RT domain.

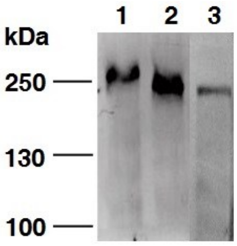

Figure S8. Western blot of cadherin fusion proteins sPgCad1-GFP (lane 1) and r15APgCad1-GFP (lane 2) and and r15BPgCad1-GFP (lane 3) produced in Hi5 cells transfected with vectors containing the *s* and *r15A* and *r15B* alleles, respectively.

1. Morin, S. *et al*. Three cadherin alleles associated with resistance to *Bacillus thuringiensis* in pink bollworm. *Proc Natl Acad Sci U S A* **100**, 5004-5009 (2003).
2. Tabashnik, B. E. et al. Sustained susceptibility of pink bollworm to Bt cotton in the United States. *GM Crops & Food* **3**, 194-200 (2012).
3. Morin, S. *et al*. DNA-based detection of Bt resistance alleles in pink bollworm. *Insect Biochem Mol Biol* **34**, 1225-1233 (2004).
4. Wang, L. *et al*. Resistance to *Bacillus thuringiensis* linked with a cadherin transmembrane mutation affecting cellular trafficking in pink bollworm from China. *Insect Biochem Mol Biol* **94**, 28-35 (2018).
