# Supplementary material for: Transposon insertion causes cadherin mis-splicing and confers resistance to Bt cotton in pink bollworm from China
Source: Sci Rep. 2019 May 16;9:7479. doi: 10.1038/s41598-019-43889-x (PMC6522560; doi:10.1038/s41598-019-43889-x)
Supplement: Supplementary file 2 — Supplementary Tables S1–5 [file 41598_2019_43889_MOESM2_ESM.pdf]

Transposon insertion causes cadherin mis-splicing and confers resistance to Bt cotton in pink bollworm from China

Supplementary Tables S1-S5

Ling Wang[1,2], Jintao Wang[1,3], Yuemin Ma[4], Peng Wan[1], Kaiyu Liu[4], Shengbo Cong[1], Yutao Xiao[5], Dong Xu[1], Kongming Wu[2*], Jeffrey A. Fabrick[6], Xianchun Li[7] & Bruce E. Tabashnik[7]

[1]Key Laboratory of Integrated Pest Management On Crops in Central China, Ministry of Agriculture, Hubei Key Laboratory of Crop Disease, Insect Pests and Weeds Control, Institute of Plant Protection and Soil Fertility, Hubei Academy of Agricultural Sciences, Wuhan 430064, China. [2]State Key Laboratory for Biology of Plant Diseases and Insect Pests, Institute of Plant Protection, Chinese Academy of Agricultural Sciences, Beijing 100193, China. [3]Hubei Insect Resources Utilization and Sustainable Pest Management Key Laboratory, Huazhong Agricultural University, Wuhan 430070, China. [4]School of Life Science, Central China Normal University, Wuhan, 430079, China. [5]Agricultural Genomics Institute at Shenzhen, Chinese Academy of Agricultural Sciences, Shenzhen, 518120, China. [6]USDA, ARS, U.S. Arid Land Agricultural Research Center, Maricopa, Arizona 85138, USA. [7]Department of Entomology, University of Arizona, Tucson, Arizona 85721, USA.

* Correspondence and requests for materials should be addressed to K.W. (E-mail address: kmwu@ippcaas.cn)

Table S1. Primers used for cloning and genotyping of *PgCad1*

Name[a]	Primer sequence (5′-3′)	Template	Strain	Size (bp)
F1	CATACTGGTGACGGTGCTTCT	cDNA	APHIS-S	2384
R1	GGACTTGGTTGTAAAGTGGGC		JL46	2384
F2	GACCTTCAGTATTCGGGAGCG	cDNA	APHIS-S	2890
R2	CATGCGCCTGTTAGTGAACTC		JL46	2587/2697
gF46	CAGGGAACTACTCACCGTTCGT	gDNA	APHIS-S	2223
gR46	ACCGTCTCCACATCGTTCAGGA		JL46	5601
*r15*F	GCAACTCATTCCGAAAACGC	gDNA	APHIS-S	None
*r15*allR1	GAGTCCACCGCCATAGAACC		JL46	652
not*r15*F	GTCCAAGGTGTGTTGAACCA	gDNA	APHIS-S	152
*r15*allR2	ATGCTGGCAGTAGGTTGTATCC		JL46	None
PgCADF	CCGGAATTCGCCACCATGGCGGGTGA<br>CGCCTGCAT	cDNA	APHIS-S	5205
PgCADR1	TCCCCGCGGACCGCCTCCGCCACCG<br>CCTGGTCGCATGCGCCTGTTAGTG	cDNA	JL46	4902
PgCADR2	TCCCCGCGGACCGCCTCCGCCACCG<br>CCCCTCTGCCGTATCTGTCTTCTC	cDNA	JL46	4182

[a]F indicates forward and R reverse.

Table S2. The CENSOR search hits of RTE-5_PGo against the Repbase

Name	From	To	Name	From	To	Class	Dir	Sim	Pos/Mm:Ts	Score
RTE-5_PGo	130	633	RTE-4_PPo	431	935	NonLTR/RTE	d	0.7628	1.9298	2197
RTE-5_PGo	634	1616	RTE-5_DPl	1719	2719	NonLTR/RTE	d	0.7652	1.7578	4520

Table S3. Responses to Cry2Ab of pink bollworm larvae from a resistant strain (JL46) and a susceptible strain (APHIS-S)

Strain	Slope (SE)[a]	LC_{50} (95% FL)[b]	RR[c]
APHIS-S	2.7 (0.3)	0.157 (0.13-0.19)	
JL46	1.8 (0.2)	0.203 (0.16-0.25)	1.3

[a]Slope of the concentration-mortality line with its standard error in parentheses.

[b]Concentration killing 50% with 95% fiducial limits in parentheses, in μg Cry2Ab per ml diet.

[c]Resistance ratio, the LC_{50} for JL46 divided by the LC_{50} for APHIS-S.

Table S4. Genetic linkage between resistance to Cry1Ac and cadherin gene *PgCad1*

	Larvae with *r15r15* (%)	
Backcross family	Control diet	Cry1Ac diet
1	47	100
2	53	100
3	43	100
4	53	100
5	48	100
Mean	49	100

PCR (Figure S4) was used to determine the genotype for a total of 258 larvae: 153 on control diet (30, 32, 30, 30 and 31 from backcross families 1-5, respectively) and 105 on diet treated with the diagnostic concentration of Cry1Ac (20, 24, 20, 20 and 21 from backcross families 1-5, respectively).

Table S5. Survival of JL46 and APHIS-S larvae on Bt cotton and non-Bt cotton

Insect strain	Cotton type	Bolls	Entry holes per boll	Survivors/boll	Survival(%) [a]	Relative survival (%)[b]
JL46	Bt	30	6.7 (0.1)	0.9 (0.0)	13.0 (0.5)	48.0 (2.0)
APHIS-S	Bt	35	5.6 (0.1)	0.0 (0.0)	0.0 (0.0)	0.0 (0.0)
JL46	Non-Bt	39	6.3 (0.1)	1.7 (0.1)	27.1 (1.1)	
APHIS-S	Non-Bt	43	5.5 (0.2)	1.7 (0.1)	31.1 (0.9)	

Values are means with their standard errors in parentheses.

[a]Larvae surviving per boll divided by entry holes per boll multiplied by 100%.

[b]Survival on Bt cotton divided by survival on non-Bt cotton.
